# Antireflection TiO_*x*_ Coating with Plasmonic Metal Nanoparticles for Silicon Solar Cells

**DOI:** 10.1007/s11468-012-9412-y

**Published:** 2012-07-07

**Authors:** Z. Starowicz, M. Lipiński, K. Berent, R. Socha, K. Szczepanowicz, T. Kruk

**Affiliations:** 1 Institute of Metallurgy and Materials Science PAS, Reymonta 25, 30-059 Kraków, Poland; 2 Institute of Catalysis and Surface Chemistry PAS, Niezapominajek 8, 30-239 Kraków, Poland

**Keywords:** Plasmonics, Photovoltaics, Silicon solar cells, Silver nanoparticles

## Abstract

It is known that the light scattering from the metal particles deposited on the surfaces of cells can be used for increasing light trapping in the solar cells. In this work, plasmonic structures are composite materials that consisted of silver nanoparticles embedded in dielectric films of TiO_*x*_—used as cell antireflection coating. The films are deposited by sol–gel method using spin-on technique. Microstructure of prepared samples is analyzed by SEM observation. Good homogenity and particles density was obtained by this simple, cheap, and short time-demanding method. We demonstrate that due to light scattering by metal particles, the plasmonic-ARC layer is more effective than TiO_*x*_ layer without Ag nanoparticles. Implementation of nanoparticles on bare cell surface was carried out too. The influence of the plasmonic structures on the silicon solar cells parameters is presented as well. We announce about 5 % additional growth in short circuit current for cells with nanoparticles.

## Introduction

Plasmonics is a new interesting branch of science with many possible applications in biology, optics, electronics, and especially in the photovoltaics. The photovoltaics is nowadays great field of science and technology with very high potential for future utilization. The two-digital percent growth of installed power all over the world has been noting for last few years. The target for plasmonics in the solar cell application is reduction of reflected and non-absorbed photons. There are few well-known methods of preventing optical losses such as antireflection coating or semiconductor surface texturization, but due to the tunable optical properties of plasmonic structures, they can be also utilized for this purpose. Main disadvantages of surface texturization are the influence on the surface recombination rate and the texture structure size. Typical pyramids are about 7 μm high, which is not acceptable in thin film solar cell technology where the total cell thickness rarely approaches few micrometers.

Various plasmonic structures can be implemented to solar cells depending on the cell technology, the materials, and processing parameters used. Three main concepts of plasmonics for photovoltaics have been presented by Atwater and Polman [1]. The first one concerns the reflection reduction by angular scattering from metal nanoparticles, which extend the optical path length, too. The second one deals with enhancement of carrier generation due to near-field effect around the particles. The third one is related to the surface plasmons polaritons propagating on the rear part of the cell. All these plasmonic effects can be used in all generations of the solar cells [2].

Recently, many authors have announced their achievements of improving solar cell efficiency by using plasmonic metal nanoparticles. The first experimental observation of light trapping was described by Stuart and Hall on silicon photodiodes. They have shown an 8-fold increase in photocurrent from silicon-on-insulator photodiodes (SOI) for wavelength of 800 nm [3]. The surface of these photodiodes was covered by 100-nm-sized silver particles deposited by vacuum evaporation. This result was an inspiration for the following researches. In the work by Schaadt [4], the nanoparticles of 80 nm in diameter were deposited on a silicon p–n junction using spherical colloidal nanoparticles of gold. The maximum enhancement of 80 % was obtained at wavelength of around 500 nm. In the work of Matheu et al. [5], an increase in conversion efficiency of 2.8 and 8.8 % from silicon solar cells respectively covered by 100 nm Au colloidal particles and 150 nm silica particles was reported.

However, the optimal plasmon structure size has been unknown for solar cells, yet. Some authors showed that too large size is not convenient for the solar cells due to multipole oscillations, which result in the *Q*_sca_ parameter decrease [2]. In the work by Temple [6], the opposite observation is presented that excitation of higher-order mode (quadrupole or higher) in large particles (>265 nm) cause the scattering in the forward direction predominately, which can be profitable for the solar cells.

The properties of plasmonic structures are dependent on material, size, shape, distribution, and refractive index of surrounding medium. Size of metal nanoparticles plays an important role. It was shown that the absorption dominates for small particles (<50 nm), while the scattering effect dominates for larger particles (≥100 nm), which is more suitable for photovoltaic applications. Dense and accurate plasmonic structures were predominantly obtained by relatively expensive method. As alternative to this, we had chosen colloidal solutions as the particle source. To place them in the destination area we applied spin coating method, which is rather uncomplicated, low-cost, and enable high throughput that is of great advantage from industrial point of view. Thus in our concept, all procedure must possess mentioned features. The antireflection layer where titanium oxide matrix is prepared by spin coating as well.

## Experiments

In order to deposit the metal particles on the solar cell surfaces, the colloidal solutions of silver nanoparticles of 70 ± 15 nm in diameter were prepared according to the procedure described elsewhere [7]. The colloid contained Ag particles suspended in water and stabilized by sodium dodecyl sulfate (SDS). The solar cells were fabricated from monocrystalline Cz–Si wafers of 76 mm diameter by industrial technology based on POCl3 diffusion and screen-printing metallization. No surface texturisation was introduced to the cells and contacts were put before antireflection coating deposition.

**Fig. 1:**
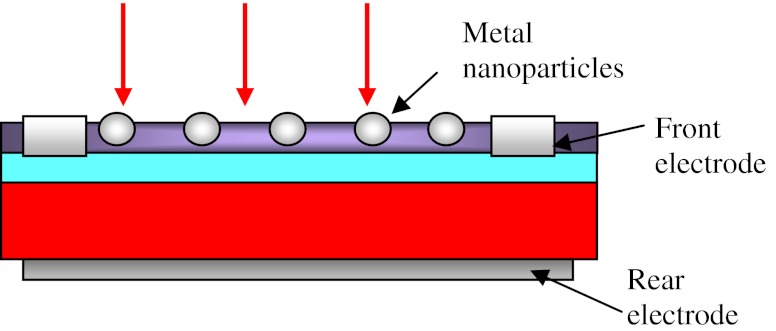
Scheme of the silicon solar cell with metal nanoparticles embedded in TiO_*x*_ layer

Our antireflection composite structure (Fig. 1) was prepared in three stages. At the first stage, silicon cell had been covered with TiO_*x*_ layer of about 40 nm. This layer was deposited by simple spin-on technique using TiO(C_2_H_5_)_4_ hydrolysis method [8], which unused in the solar cell industry yet. Then the TiO_*x*_ coating was heated for solidification at the temperature of 200°C for 10 min. In the next step, the Ag particles were deposited by injecting liquid on spinning wafer. Finally, the second TiO_*x*_ layer was deposited in order to form the ARC layer with sufficient thickness of about 80 nm with embedded plasmonic particles. For comparison, silver nanoparticles were deposited on the front surface of the bare cell without any antireflection coating as well. At the end, the cells were characterized by current–voltage measurement in order to find the influence of plasmonic nanoparticles on the electrical parameters.

The samples were analyzed by scanning electron microscopy (SEM) using backscattered electrons (BSE) imaging. The magnification required to identify nanoparticles was ×40,000. The images were obtained at the accelerating voltage of 20 kV and the 10 mm working distance. The element identification was obtained by Energy Dispersive Spectrometry (EDS). The SEM-EDS analysis was performed on FEI E-SEM XL 30 microscope with the EDAX GEMINI 4000 energy dispersive spectrometer.

## Results and Discussion

**Fig. 2:**
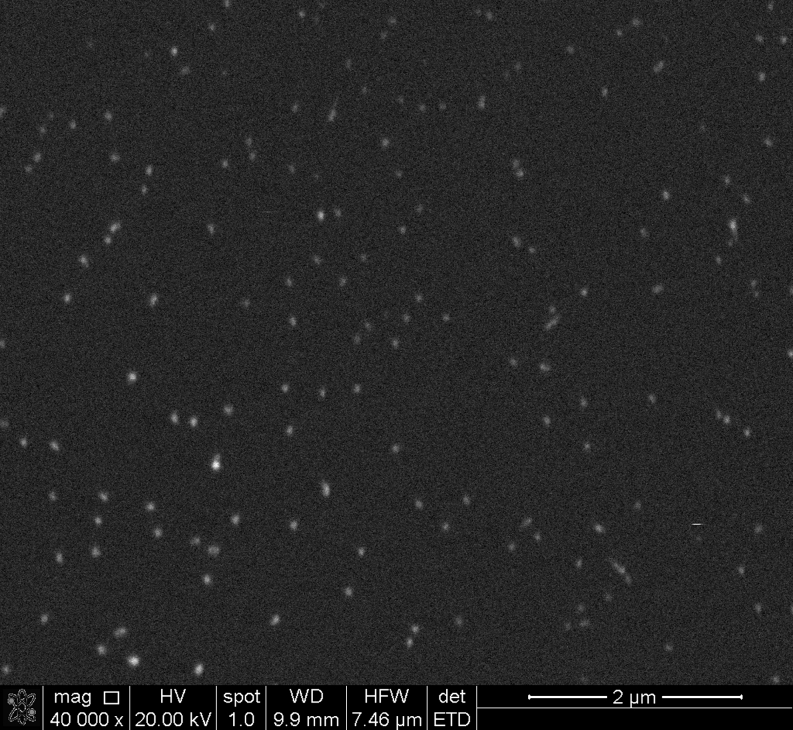
SEM image of the silver nanoparticles placed on TiO_*x*_ layer covering Si wafer

The SEM image of particles distribution at the surface is shown in Fig. 2. The BSE yield of high atomic number (Z) increased. Elements as Ag particles can be compared to low density matrix. The EDS elemental analysis confirmed further that these bright spots were Ag. Their size was of about 70 nm. The EDS microanalysis showed also a uniform distribution of titanium and oxygen. The measured particle density was about 3·10^8^/cm^2^.

**Fig. 3:**
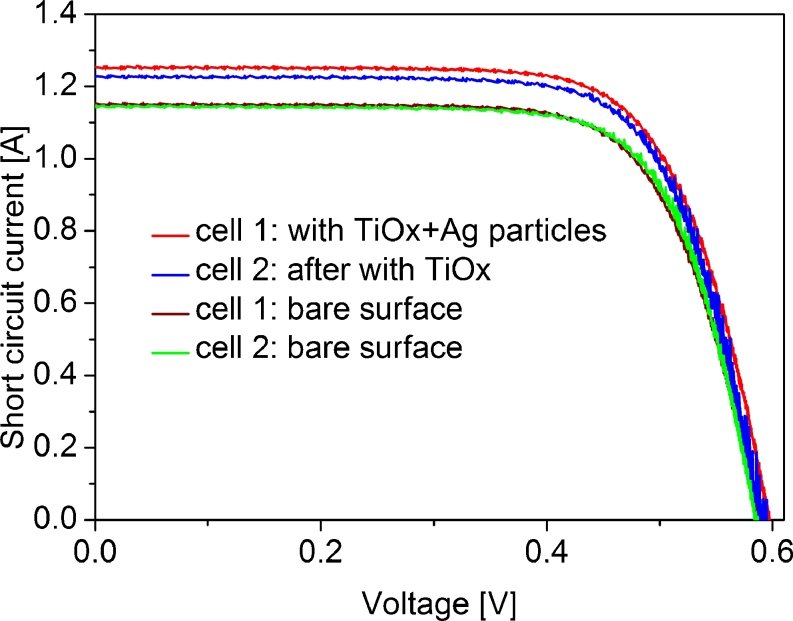
Current–voltage characteristics of the solar cells with and without silver nanoparticles embedded in the TiO_*x*_ layer and the cells with bare surface

The determination of electrical parameters of the cell revealed high increase of short circuit current and energy conversion efficiency, as it was expected (Table 1, Fig. 3). The cell coated with pure TiO_*x*_ layer increased the *I*_SC_ from 940 to 1,227 mA, which accounts to the growth of about 30 %. The cell with Ag nanoparticles embedded in TiO_*x*_ resulted in *I*_SC_ increase of about 35 %, i.e., from 928 to 1,253 mA. This additional 5 % growth of short circuit current can be ascribed to plasmonic nanoparticles that scattered more photons to the substrate in comparison to the particle free cell.

**Table 1:** Electrical parameters of 3-in. diameter solar cell coated with TiO_*x*_ and TiO_*x*_ + Ag layers compared with the bare one

Cell type	*I*_SC_ [mA]	Voc [mV]	FF [%]	Eff [%]
Cell 1—bare	928	578	73	8.9
Cell 1—with Ag + TiO_*x*_	1,253	599	71	12.1
Cell 2—bare	940	576	74	9.1
Cell 2—with TiO_*x*_	1,227	590	72	11.8

Influence of the Ag nanoparticles on the efficiency of cell formed without any antireflection coating was also studied. It was found that deposition of nanoparticles at the cell surface also resulted in improvement of the cell parameters. For this system, the measured enhancement was about 5.4 %.

## Summary

The simple deposition method of composite antireflection TiO_*x*_ coating improved by embedded plasmonic silver nanoparticles has been described. The high-density distribution of uniformly sized (about 70 nm) particles was obtained from colloidal solution by spin-on method. Our experiments confirmed the potential of application of plasmonic nanostructures for industrial silicon solar cells manufacturing. Additional increase of short-circuit current of about 5 % in the solar cells with TiO_*x*_ + Ag composite antireflection coating was approached.

## References

[1] Atwater HA, Polman A (2010). Plasmonics for improved photovoltaic devices. Nat Mater.

[2] Pillai S, Green MA (2010). Plasmonics for photovoltaic applications. Sol Energ Mater Sol Cell.

[3] Stuart R, Hall DG (1998). Island size effect in nanoparticles enhanced photodiodes. Appl Phys Lett.

[4] Schaadt DM, Feng B, Yu ET (2005). Enhanced semiconductor optical absorption via surface plazmon excitation in metal nanoparticles. Appl Phys Lett.

[5] Matheu P, Lim SH, Derkacs D, McPheeters C, Yu ET (2008). Metal and dielectric nanoparticles scattering for improved optical absorption in photovoltaic devices. Appl Phys Lett.

[6] Temple TL, Mahanama GDK, Reehal HS, Bagnall DM (2009). Influence of localized surface plazmon excitation in silver nanoparticles on the performance of silicon solar cells. Sol Energ Mater Sol Cell.

[7] Szczepanowicz K, Stefańska J, Socha RP, Warszyński P (2010). Preparation of silver nanoparticles via chemical reduction and their antimicrobial activity. Physicochem Probl Miner Proces.

[8] Lipiński M, Żdanowicz T (1992). Optimization of the optical parameters of TiO_*x*_ ARC layers deposited by spin-on technique. Sol Energ Mater Sol Cell.

